# Exploring the key genetic association between chronic pancreatitis and pancreatic ductal adenocarcinoma through integrated bioinformatics

**DOI:** 10.3389/fgene.2023.1115660

**Published:** 2023-07-12

**Authors:** Kai Guo, Yatong Zhao, Yingying Cao, Yuan Li, Meng Yang, Ying Tian, Jianmeng Dai, Lina Song, Shuai Ren, Zhongqiu Wang

**Affiliations:** ^1^Department of Radiology, Affiliated Hospital of Nanjing University of Chinese Medicine, Nanjing, China; ^2^ School of Medicine, Tongji University, Shanghai, China

**Keywords:** pancreatic ductal adenocarcinoma, chronic pancreatitis, bioinformatics, CEL, WCGNA, differential gene analysis

## Abstract

**Background:** Pancreatic ductal adenocarcinoma (PDAC) develops rapidly and has a poor prognosis. It has been demonstrated that pancreatic ductal adenocarcinoma and chronic pancreatitis (CP) have a close connection. However, the underlying mechanisms for chronic pancreatitis transforming into pancreatic ductal adenocarcinoma are still unclear. The purpose of this study was to identify real hub genes in the development of chronic pancreatitis and pancreatic ductal adenocarcinoma.

**Methods:** RNA-seq data of chronic pancreatitis and pancreatic ductal adenocarcinoma were downloaded from the Gene Expression Omnibus (GEO) database. Weighted gene co-expression network analysis (WGCNA) was performed to construct a gene co-expression network between chronic pancreatitis and pancreatic ductal adenocarcinoma. GEO2R and a Venn diagram were used to identify differentially expressed genes. Then visualized networks were constructed with ClueGO, and modules of PPI network were calculated by MCODE plugin. Further validation of the results was carried out in two additional cohorts. Analyses of *CEL*-coexpressed genes and regulators including miRNAs and transcription factors were performed by using the corresponding online web tool. Finally, the influence of *CEL* in the tumor immune microenvironment (TIME) was assessed by immune contextual analysis.

**Results:** With the help of WGCNA and GEO2R, four co-expression modules and six hub genes were identified, respectively. ClueGO enrichment analysis and MCODE cluster analysis revealed that the dysfunctional transport of nutrients and trace elements might contribute to chronic pancreatitis and pancreatic ductal adenocarcinoma development. The real hub gene *CEL* was identified with a markedly low expression in pancreatic ductal adenocarcinoma in external validation sets. According to the miRNA-gene network construction, hsa-miR-198 may be the key miRNA. A strong correlation exists between *CEL* and TIME after an evaluation of the influence of *CEL* in TIME.

**Conclusion:** Our study revealed the dysfunctional transport of nutrients and trace elements may be common pathogenesis of pancreatic ductal adenocarcinoma and chronic pancreatitis. Examination on these common pathways and real hub genes may shed light on the underlying mechanism.

## Introduction

According to the latest global cancer statistics, pancreatic ductal adenocarcinoma (PDAC) is the third leading cause of cancer-related death worldwide (Siegel et al., 2023). In the United States, PDAC is predicted to be the second leading cause of cancer-related deaths by 2030 (Rahib et al., 2014). At present, PDAC is difficult to diagnose at an early stage due to its hidden location, lack of specific symptoms, and aggressive behavior, which often delays effective treatment. Therefore, it is imperative to develop tools for detecting early-stage PDAC before it spreads beyond the pancreas and progresses into a late stage. Chronic pancreatitis (CP) is a spectrum of fibro-inflammatory disease that causes progressive damage to the parenchyma of the pancreas. It is well recognized that CP is a risk factor for PDAC (Monroy-Iglesias et al., 2021). A large retrospective cohort study found that patients with CP had a 14-fold increased risk of developing PDAC (Lowenfels AB et al., 1993). Around one in six patients was diagnosed with PDAC within 2 years of being diagnosed with CP (Kirkegard et al., 2017). Pancreatic acinar cell metaplasia is observed when CP progresses to PDAC (Yang et al., 2022). Inflammation-associated signals regulate PDAC progression and therapeutic resistance by promoting aberrant cell proliferation, metastasis, and inhibiting apoptosis (Hausmann et al., 2014). Although more and more studies have substantiated a significant link between CP and PDAC, genetic research in this area is still limited and needs to be explored.

In recent years, bioinformatics has become a growing research hotspot for analyzing gene expression profiles, and comorbidity networks can be used to analyze multimorbidity (Su et al., 2021; Yao et al., 2021). Employing two original microarray datasets obtained from the Gene Expression Omnibus (GEO) database, we investigated co-expression modules and differentially expressed genes (DEGs) between CP and PDAC in the present study. As a result, we found the *CEL* to be the real hub gene related to CP and PDAC, and the dysfunctional transport of nutrients and trace elements might play a pivotal role in the development of CP and PDAC. Additionally, the role of *CEL* in the tumor immune microenvironment (TIME) of PDAC has also been identified. As far as we know, this may be the first time to perform comorbidity analysis to explore the real hub gene and its characteristics in the development of CP to PDAC.

## Materials and methods

### Gene expression profile data collection

Gene expression profiles were downloaded from the GEO database using the search terms “chronic pancreatitis” and “pancreatic ductal adenocarcinoma.” Following criteria were used to screen the obtained datasets: First, all sequencing data were obtained from human pancreatic tissue; Second, no patients had received any chemoradiotherapy prior to the surgical resection; Third, WGCNA accuracy requires at least 10 samples in each discovery group. Finally, the four GEO datasets numbered GSE143754, GSE91035, GSE101462 and GSE151945 were selected for further analysis. ACLBI database (https://www.aclbi.com/static/index.html#/) and R software (version 3.4.1) were used to normalize microarray data using the normalize.quantiles function of the preprocessCore package. Then normalized data on the platform were used to convert probes to gene symbols. Raw data and code are available in a GitHub repository (https://github.com/KaiGuo2023/KaiGuo-CP-PDAC.git).

### Weighted gene co-expression network analysis

Weighted networks were adopted to quantify network connections by assigning connection strengths to gene pairs. In this study, a weighted gene co-expression network analysis (WGCNA) package (https://cloud.oebiotech.cn/task/detail/wgcna-oehw/) was used to analyze the association between gene modules and traits in CP and PDAC (Langfelder and Horvath, 2008). Gene co-expression networks are scale-free weighted gene networks. To meet the scale-free network distribution preconditions, a power value for the adjacency matrix weight parameter must be chosen. Based on the power value set between 1 and 30, the average connectivity and correlation coefficient for the network were calculated. The higher the correlation coefficient (maximum of 1), the closer network is to a scale-free distribution. And at the same time, gene connectivity must be maintained. The DEGs were then divided into several modules by using the selected power values based on a weighted co-expression network model. An analysis of clustering dendrograms revealed the results of dynamic tree cutting and merging. The module structure was visualized through a heat map and a topological overlap matrix (TOM) plot. A *Pearson* correlation algorithm was used to calculate the correlation coefficient and *p*-value of module characteristic genes and traits. Modules associated with individual traits were screened at a threshold of 0.3 correlation coefficient and a *p*-value of 0.05. Finally, the “Module Eigengenes” algorithm was used to determine the eigengene values of specific modules, and the gene expression profiles of individual modules were summarized. For each trait-associated module, we calculated correlations (GS, Gene Significance) between module gene expression and corresponding traits separately, as well as correlations (MM, Module Membership) between module gene expression and module eigengenes. Scatter plots were obtained in accordance with the above two values.

### Detection of real hub genes in CP and PDAC

These modules were selected because they were highly relevant to CP and PDAC, respectively (red, cyan, black and lightcyan). The intersection of these genes with high correlation coefficients was then identified and visualized with the online Venn diagram tool (https://hiplot-academic.com/basic/venn). The DEGs were screened out with the GEO2R online tool (https://www.ncbi.nlm.nih.gov/geo/geo2r/). The DEGs were identified by multiplying | log_2_(fold change) | by 1 and adjusting *p*-values by 0.05 for each fold change. With fold change values and *p*-adjustments, a volcano plot was constructed. Subsequently, Venn diagrams were used to visualize the consistently regulated genes among the significant DEGs. A subsequent analysis of these overlapping DEGs was conducted.

ClueGO, a Cytoscape plug-in, was used to perform functional enrichment analysis on shared genes to determine their biological relevance. PPI networks have a topological property that allows investigation of key genes through Metascape (http://metascape.org), and a confidence score >0.7 was set as the cut-off value. The Molecular Complex Detection (MCODE) tool in Metascape was used to identify highly interconnected unique clusters in CP and PDAC. Then, the DAVID tool (https://david-d.ncifcrf.gov/) was used to analyze biological processes among overlapping DEGs. The cytoHubba of Cytoscape was conducted to score each node gene by the top 7 algorithms (MCC, MNC, Degree, EPC, Closeness, Radiality, and Stress). Upset plots were subsequently drawn with the above scores to visualize the intersections across overlapping gene sets. Real hub genes were derived by taking the intersection of hub genes in WCGNA co-expression network and hub genes in PPI network, and the differences between two groups at each gene of interest were visualized with box plots.

### Validation of real hub genes through DEGs analysis

DEGs analysis was carried out using additional CP and PDAC data sets (GSE101462 and GSE151945) to verify the hub genes in these two entities. DEGs between CP and PDAC samples were identified using the ACLBI database (*p* < 0.05). Meanwhile, GO and KEGG databases were used to annotate DEGs. The mutation data of above real hub gene was obtained from TCGA at cBioPortal Cancer Genomics (http://www.cbioportal.org/public-portal/index.do). We compared the expression levels of real hub gene in PDAC and normal tissues using GEPIA database (http://gepia.cancer-pku.cn). A validation of the immunohistochemistry of the real hub gene was also conducted using the Human Protein Atlas (HPA) database (http://www.proteinatlas.org/). Clinical information of the 178 patients with PDAC was available in the TCGA database (https://tcga-data.nci.nih.gov/tcga/). According to real hub gene median expression, clinical data were extracted from the TCGA database and divided into high and low mRNA expression groups. Baseline patient characteristics and presenting clinical features were summarized according to Xiantao tool (https://www.xiantao.love/products). Logistic regression analysis was used to determine the diagnostic performance of real hub gene in the differential diagnosis between TCGA and GTEx normal pancreas samples and PDAC samples.

### Interactions between *CEL* and its co-expressed genes

To determine which genes were co-expressed with *CEL*, the LinkedOmics database (http://www.linkedomics.org) was used to analyze the transcriptome data of PDAC from the TCGA (Vasaikar et al., 2018). The online analysis tool of this database produced a volcano plot of genes positively and negatively correlated with *CEL* expression and a heatmap of the top 50 positively/negatively related genes. Based on the DAVID database, GO and KEGG analyses were performed for the *CEL* co-expression genes with respect to biological process (BP), cellular component (CC), molecular function (MF), and KEGG pathway.

### Predicting upstream miRNAs and transcription factors of *CEL*

It has been demonstrated that microRNAs (miRNAs) can influence gene expression by inhibiting translation or promoting mRNAs degradation (Correia de Sousa et al., 2019). Therefore, a further investigation was conducted on whether some miRNAs were involved in regulating risk genes in CP and PDAC. HMDD (http://cmbi.bjmu.edu.cn/hmdd) was used to obtain human miRNA and miRNA-disease association data. The miRNA-target relationships were obtained from miRWalk (http://mirwalk.umm.uni-heidelberg.de/). To construct the gene-TF regulatory network, the Transcriptional Regulatory Relationships Unraveled by Sentence-based Text mining (TRRUST, http://www.grnpedia.org/trrust/) database was used to identify the transcription factors (TFs) of the real hub gene between CP and PDAC.

### Assessing the landscape of tumor immune microenvironment

Immune cells play a fundamental role in the development of diseases. With the R package “estimate” and Xiantao online tool, immune and stromal components of each sample were determined. A single sample gene-set enrichment analysis (ssGSEA) score based on 24 immune-associated gene sets was referred to assess the enrichment level and activity of several immune cells in PDAC. Through the deconvolution method, TIMER provides estimates of the levels of tumor-infiltrating immune cells, including B cells, CD8^+^ T cells, CD4^+^ T cells, macrophages, neutrophils, and dendritic cells (https://cistrome.shinyapps.io/timer/). TCGA RNA-seq data were retrieved to evaluate eight immune checkpoint-related genes. The Wilcoxon rank sum test or the Kruskal–Wallis test was used for two-sample or multiple-sample group comparisons, respectively. TISIDB database (http://cis.hku.hk/TISIDB/index.php) was deployed to explore the correlation between *CEL* expression and immune subtypes and immunomodulators in PDAC. TIDE algorithm was used to predict potential ICB responses. The survival analyses were conducted using the Kaplan-Meier (KM) method to examine the relationships between immune subgroups and clinical characteristics, and the log rank tests were used to detect the differences between them.

## Results

### Information for GEO database

On the basis of the prior criteria, the four GEO datasets numbered GSE143754, GSE91035, GSE101462 and GSE151945 were selected for further analysis. Table 1 summarizes the information of the four datasets, including GSE number, platform, sample, and type of groups. WGCNA was performed on GSE143754 and GSE91035, followed by validation on the remaining two sets.

**TABLE 1:** Summary of GEO datasets containing the CP/PDAC patients.

ID	GSE number	Platform	Samples	Group
1	GSE143754	GPL17586	6 CP patients and 9 controls	Discovery
2	GSE91035	GPL22763	25 PDAC patients and 8 controls	Discovery
3	GSE101462	GPL10558	10 CP patients and 4 PDAC patients	Validation
4	GSE151945	GPL17077	3 CP patients and 3 PDAC patients	Validation

### Co-expression modules in CP and PDAC

**FIGURE 1:**
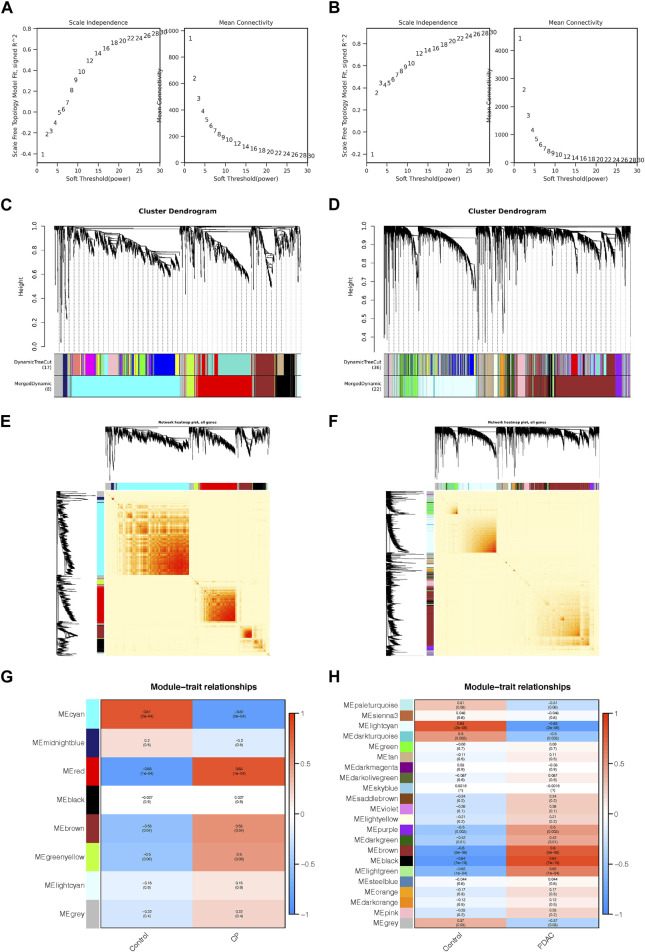
Co-expression networks constructed for CP and PDAC datasets. **(A, B)** Network topology analysis of different adjacency matrix weight parameter power. As shown on the left, the power affected the scale-free topology fit index. And in the right diagram, the power affected mean connectivity. **(C, D)** Genome dendrograms obtained by hierarchical clustering using mean linkage. Below the dendrogram were colored rows showing the allocation of modules determined by Dynamic Tree Cutting. **(E, F)** All genes were shown as a heat map plot with their topological overlap matrix (TOM). Red indicated higher overlap, while light indicated low overlap. Gene dendrograms and module assignments were shown on the left side and top side, respectively. **(G, H)** Heap of module-trait relationships in CP **(G)** and PDAC **(H)**. CP, chronic pancreatitis; PDAC, pancreatic ductal adenocarcinoma.

For the construction of a scale-free network in CP and PDAC, the adjacency matrix weight parameter power was selected to be 30 and 20 respectively (Figures 1A, B). A weighted co-expression network model was constructed based on selected power values. Finalized in CP, 1773 genes were divided into 8 modules (Figure 1C), while 8,632 genes in PDAC were divided into 22 modules (Figure 1D), of which the gray module (gray) consisted of genes that could not be assigned to any module without any reference significance. After analyzing gene module interactions, hierarchical clustering dendrograms and modules were used to generate Tom plots of gene networks (Figures 1E, F). The genes with similar expression patterns were combined into the same module. The *Y*-axis represents the level of intramolecular connectivity to genes at the top of the module branch that have greater connectivity to other genes within the module. Using hierarchical clustering and correlation analyses, the correlations between the modules and phenotypes (control and CP/PDAC) were calculated (Figures 1G, H). There were two modules “red” and “cyan” with a high association with CP (red module: r = 0.83, *p* = 1e-04; cyan module: r = −0.81, *p* = 2e-04). Moreover, a total of 22 modules were identified in GSE91035. The “black” (r = 0.84, *p* = 7e-10) and “light cyan” (r = −0.83, *p* = 2e-09) modules were highly associated with PDAC.

**FIGURE 2:**
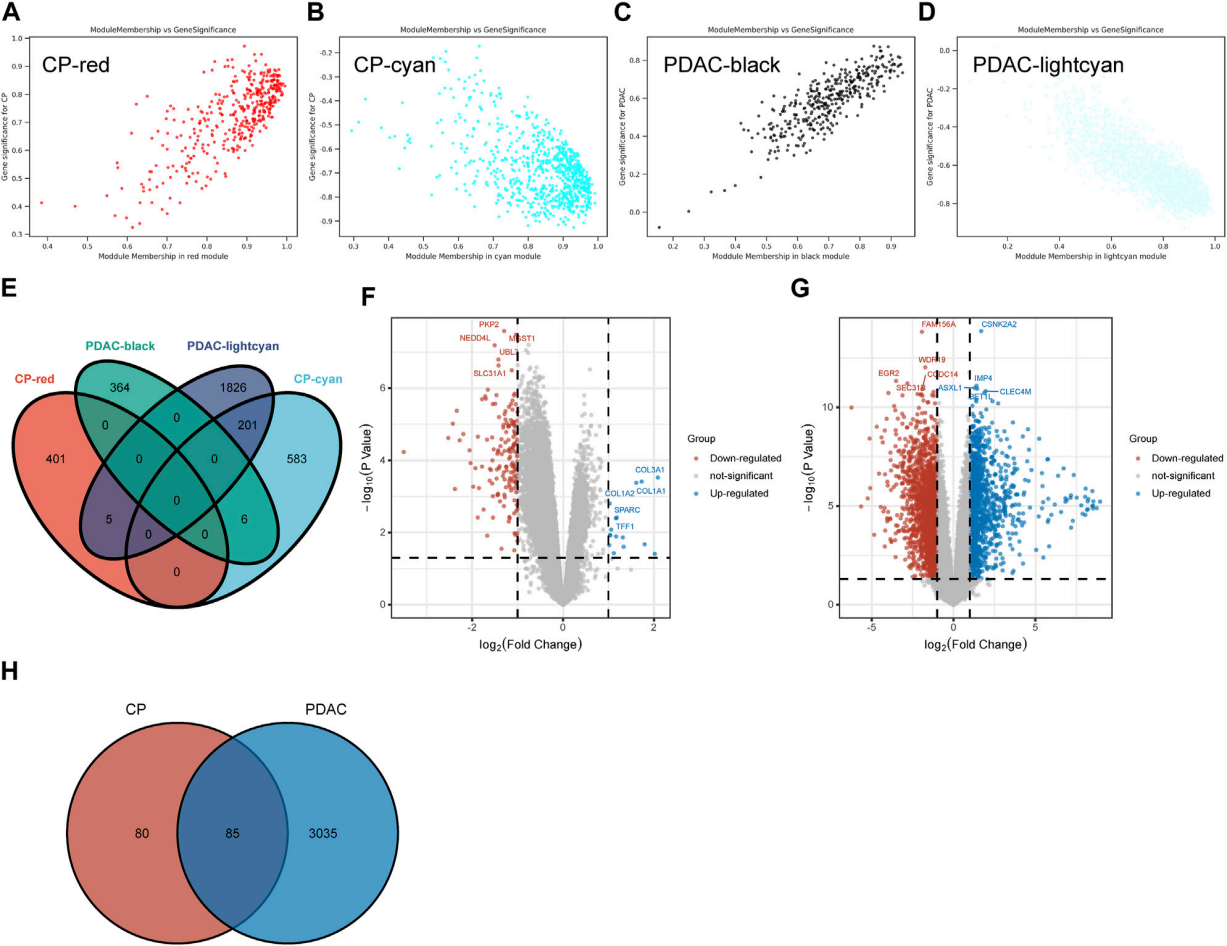
The Shared Genes in CP and PDAC. **(A–D)** Scattered plots were generated from the results of GS and MM. MM values in the module correspond to the abscissa, GS values for each gene in the trait correspond to the ordinate, and each point in the picture corresponds to one gene in the module. **(E)** Venn diagram of the shared genes between the two CP modules and two PDAC modules. **(F)** Volcano map of GSE143754. **(G)** Volcano map of GSE91035. Upregulated genes were marked in blue; downregulated genes were marked in red. The figure shows two vertical dashed lines, representing log_2_ (FC) at −1 and 1; Horizontally dashed line represents adjusted *p*-value at 0.05. **(H)** Venn diagram of DEGs in GSE143754 and GSE91035 gene chips. GS, Gene Significance; MM, Module Membership; DEGs, differentially expressed genes.

A total of 406 genes were included in the red module, which displayed a positive correlation with CP (Figure 2A). There were 790 genes in the cyan modules that were negatively correlated with CP (Figure 2B). In GSE91035, black and light cyan module including 370 and 2032 genes, respectively (Figures 2C, D). A total of 212 genes were shared in the highly correlated modules of CP and PDAC, and this component of the overlap was closely related to their pathogenesis (Figure 2E). Microarray results were standardized and DEGs (165 in GSE143754 and 3,120 on GSE91035) were identified (Figures 2F, G). In the intersection of the Venn diagram, 85 overlapping DEGs were identified (Figure 2H).

### The unique gene signatures of CP and PDAC

**FIGURE 3:**
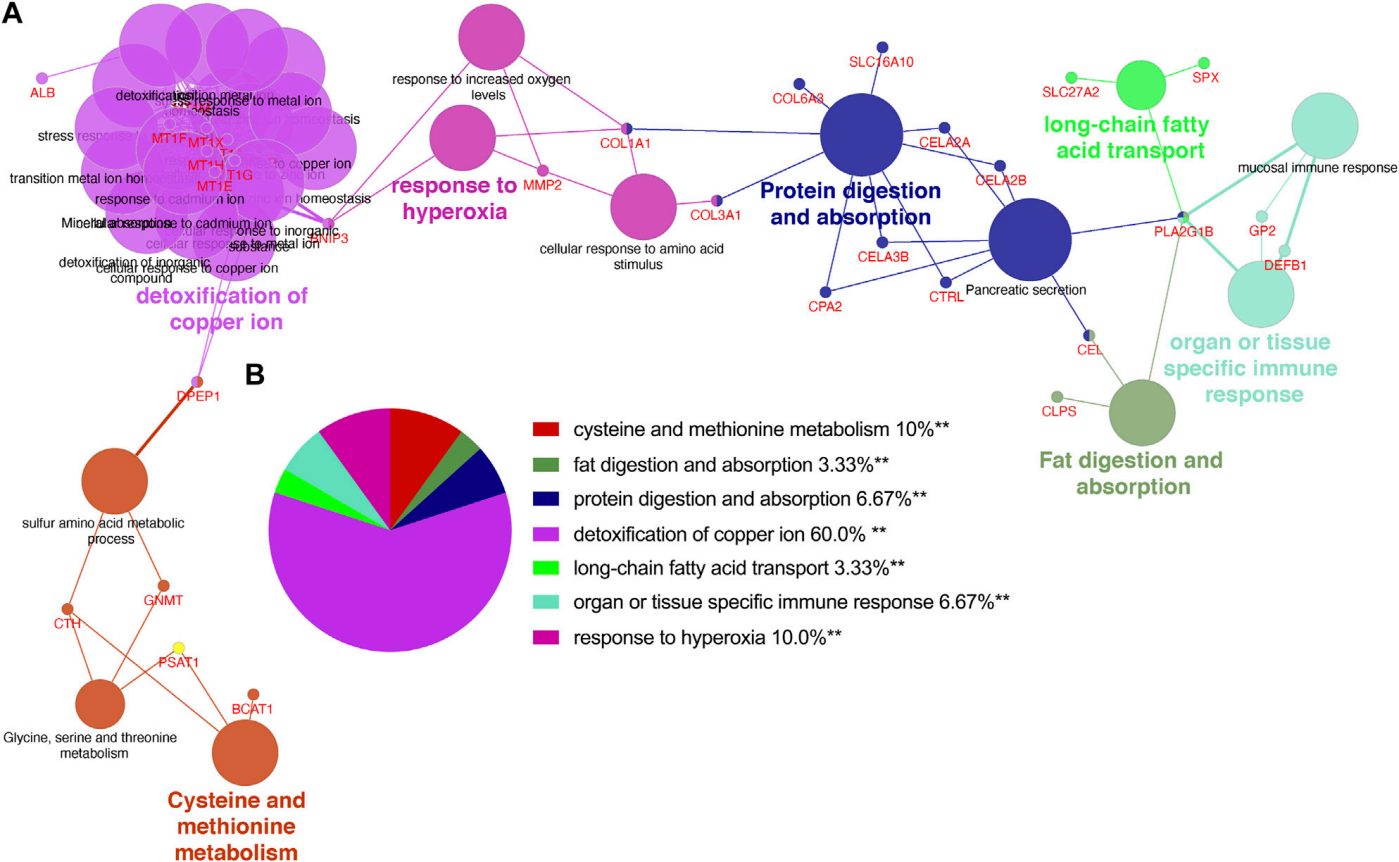
The enrichment analysis was performed by ClueGO tool. **(A)** Interaction network between GO terms (different pathways were represented by different colors). **(B)** Pie chart showed the percentage of GO terms in the shared genes. GO, gene ontology.

Utilizing ClueGo, we analyzed the GO enrichment of 212 selected shared genes to explore their potential functions. The results revealed that these genes were enriched in detoxification of copper ion, cysteine and methionine metabolism, response to hyperoxia, protein digestion and absorption, organ or tissue specific immune response, fat digestion and absorption and long-chain fatty acid transport (Figure 3A). Detoxification of copper ion accounted for 60% of total GO terms, indicating its importance in both CP and PDAC. “Cysteine and methionine metabolism” and “response to hyperoxia” ranked second at the same time, and each accounted for 10%. Besides, “protein digestion and absorption” and “organ or tissue specific immune response” accounted for 6.67%, respectively. “Fat digestion and absorption” and “long-chain fatty acid transport” also accounted for 3.33%, respectively (Figure 3B).

**FIGURE 4:**
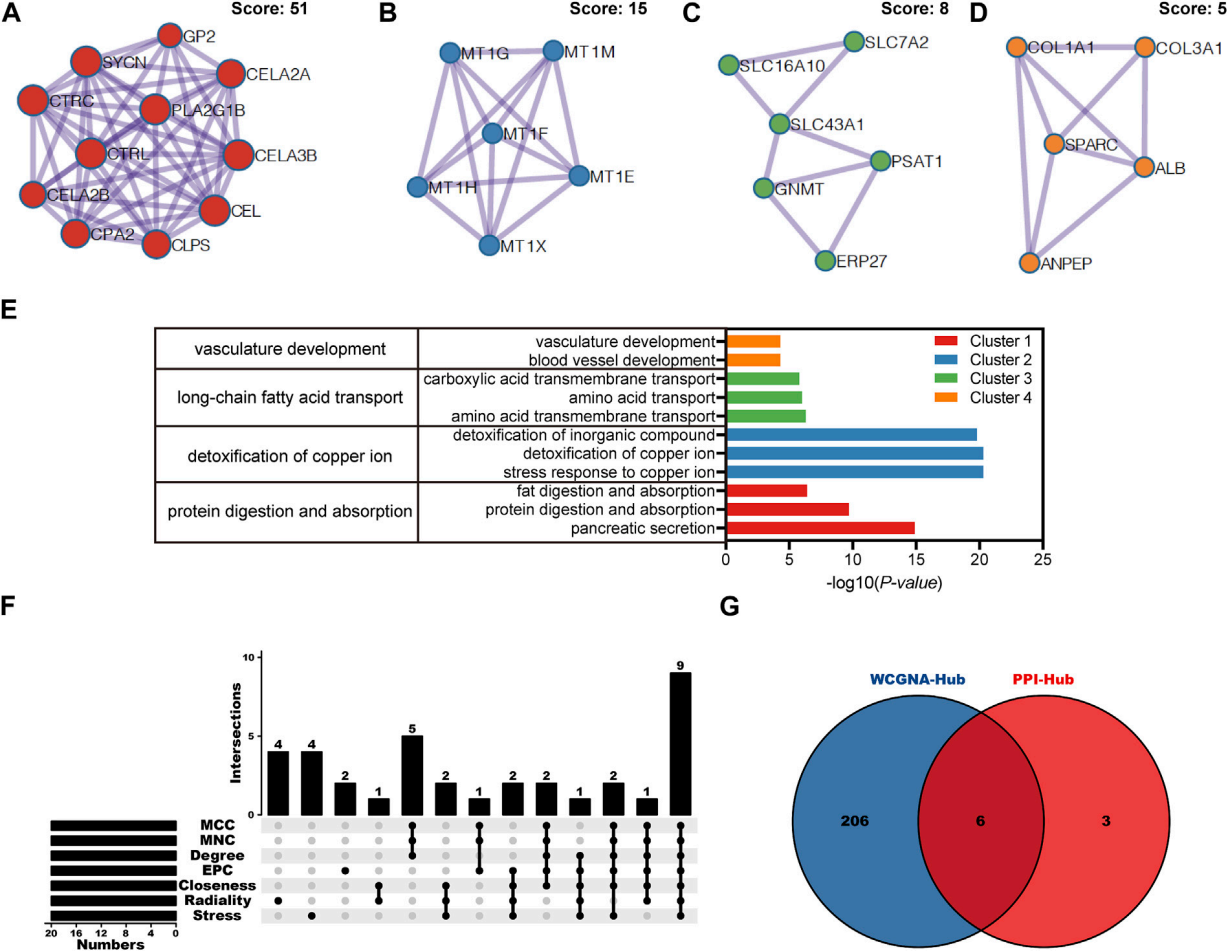
PPI network, module analysis, and hub gene identification. **(A–D)** Four module analyses constructed from the PPI network. **(E)** GO biological process of the four clusters. **(F)** Upset diagram identified nine most critical genes that were shared by seven feature selection algorithms. **(G)** Venn diagram presented real hub genes under WCGNA co-expression and those involved in the PPI network. PPI, Protein-protein interactions; GO, gene ontology.

Based on above 85 overlapping DEGs data, 4 clusters were created using MCODE algorithm (confidence score >0.7 was set as the cutoff) in Metascape. Cluster 1 consisted of 11 nodes (score: 51) (Figure 4A); Cluster 2 consisted of 6 nodes (score: 15) (Figure 4B); Cluster 3 consisted of 6 nodes (score: 8) (Figure 4C); Cluster 4 consisted of 5 nodes (score: 5) (Figure 4D). Functional annotation with DAVID was carried out for each gene cluster. According to functional enrichment analysis, the 4 clusters mainly involved in protein digestion and absorption, detoxification of copper ion, long-chain fatty acid transport, as well as in vasculature development (Figure 4E). A total of seven algorithms were then used to calculate the gene score for each node. In Figure 4F, 9 hub genes were identified with boxes by using R package “UpSet.” A total of 6 hub genes (*ALB, CEL, CELA3B, CTRL, PLA2G1B* and *SYCN*) were selected for further validation analysis from both the WCGNA co-expression network and PPI networks (Figure 4G; Supplementary Figure S1).

### Validation of the expression of hub genes

**FIGURE 5:**
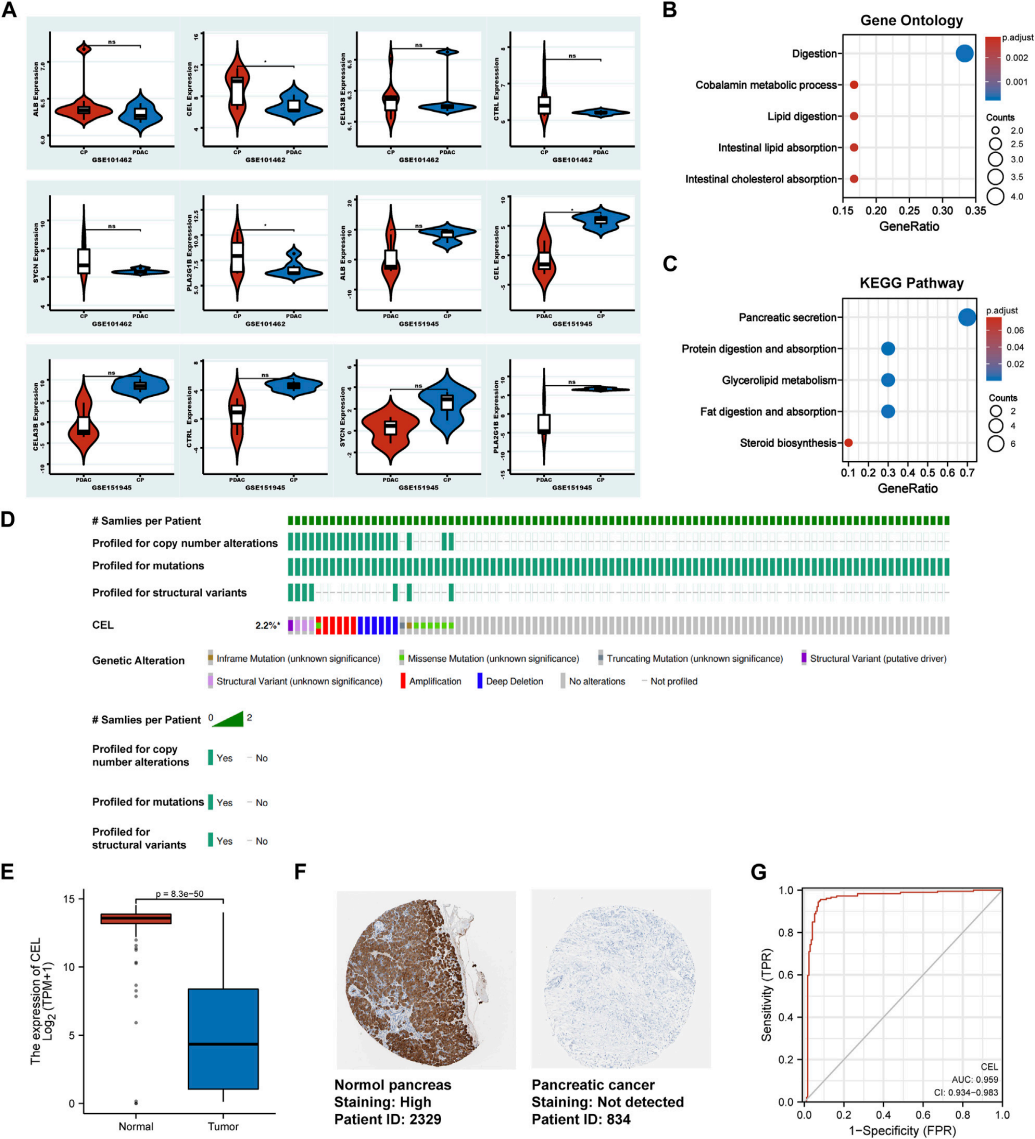
Identification of the real hub DEGs. **(A)** Boxplots of the expression of real hub genes in validation sets. **(B, C)** The DEGs from validation sets were employed for GO and KEGG analysis, respectively. **(D)** The mutation information for *CEL* gene in cBioPortal. **(E)** Boxplots showed the mRNA expression level of *CEL*. Red represents normal, blue represents tumor. **(F)** The *CEL* protein expression level in the HPA database. **(G)** ROC curve of *CEL* relative expression for diagnosis of PDAC. The abscissa was the false positive rate, and the ordinate was the true positive rate. DEGs, differentially expressed genes; GO, Gene Ontology; KEGG, Kyoto Encyclopedia of Genes and Genomes; HPA, Human Protein Atlas; PDAC, pancreatic ductal adenocarcinoma.

In validation cohorts (GSE101462 and GSE151945), we conducted differential gene analysis between CP and PDAC to validate real hub gene. It can be seen from the results that *CEL* gene exhibited significant differences in expression at the both validation sets (*p* < 0.05). And the expression of *ALB, CELA3B, CTRL, PLA2G1B* and *SYCN* genes between CP and PDAC groups did not simultaneously exhibit statistical differences in validation set (Figure 5A). So, the real hub gene carboxyl ester lipase (*CEL*), also known as bile salt-dependent lipase (*BSDL*), which may play a pivotal role in both CP and PDAC. Gene Ontology (GO) and Kyoto Encyclopedia of Genes and Genomes (KEGG) pathways were used to analyze the DEGs in validation cohorts respectively. For GO enrichment analysis, it can be found that the DEGs were mainly enriched in “Digestion,” “Cobalamin metabolic process,” “Lipid digestion,” “Intestinal lipid absorption” and “Intestinal cholesterol absorption” (Figure 5B). The KEGG pathway enrichment results showed that the DEGs were enriched in “Pancreatic secretion,” “Protein digestion and absorption,” “Glycerolipid metabolism,” “Fat digestion and absorption,” and “Steroid biosynthesis” (Figure 5C). In TCGA tumor samples, *CEL* mutation status was analyzed using the cBioPortal tool. The CBioPortal tool indicates that about 2.2% of pancreatic cancer patients have *CEL* gene mutations. Missense mutation and deep deletion were the most common *CEL* variant type (Figure 5D). In PDAC tissues, the GEPIA database showed significantly low expression of *CEL* mRNA (Figure 5E). Furthermore, the CEL protein expression was explored using HPA database. The typical immunohistochemistry result revealed downregulated CEL expression in PDAC tissues (Figure 5F). *CEL* gene was highly accurate in predicting the outcome of both normal and PDAC tissue (AUC = 0.968, CI = 0.947–0.990) (Figure 5G). A total of seven clinical features were analyzed based on the TCGA database (age, gender, T stage, N stage, M stage, pathologic stage and anatomic neoplasm subdivision). As the result suggested (Supplementary Table S1), alteration in gene was significantly associated with N stage (*p* = 0.010), pathologic stage (*p* = 0.007), and anatomic neoplasm subdivision (*p* = 0.02).

### *CEL* Co-expression networks and enrichment analyses

**FIGURE 6:**
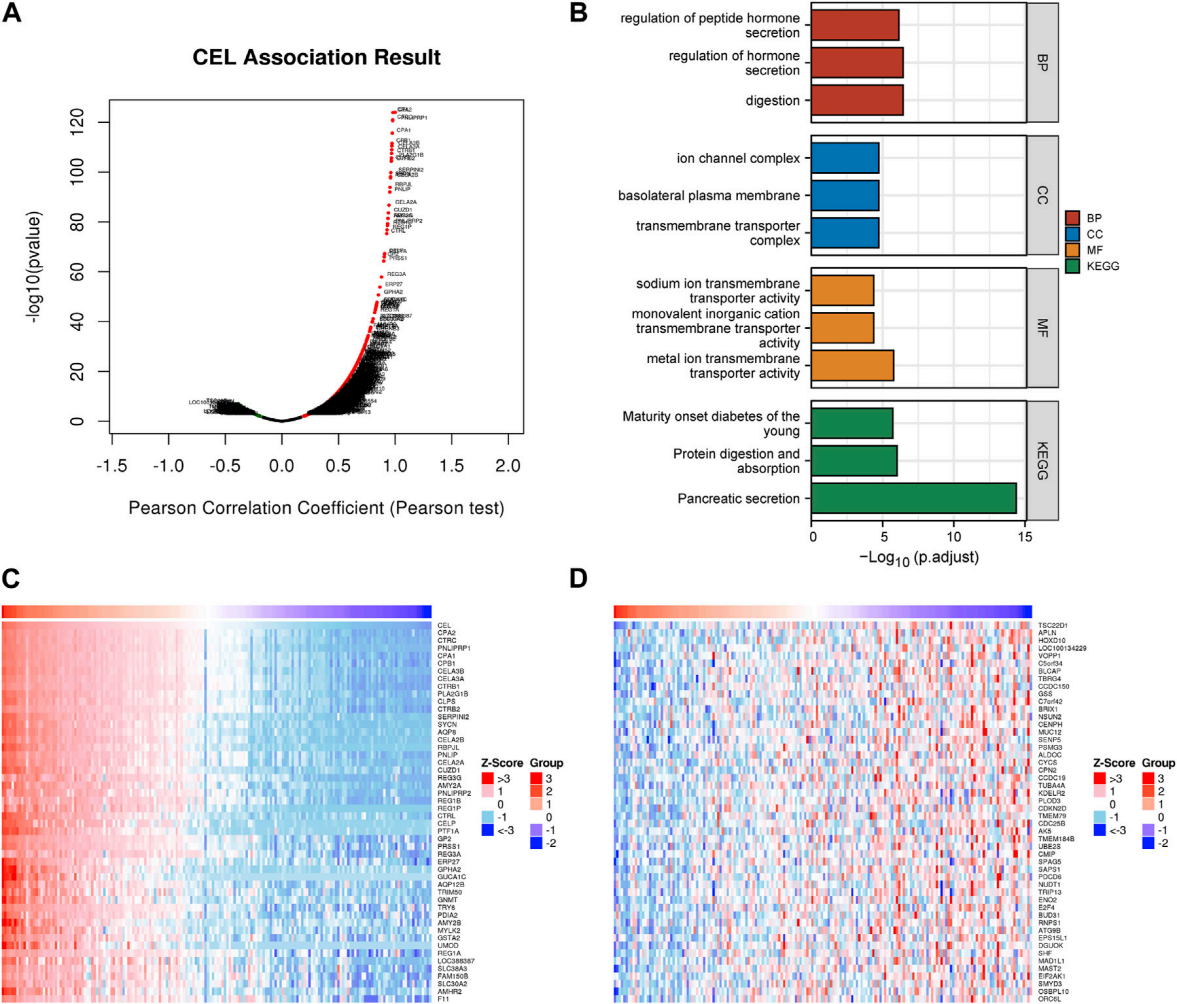
Co-expression gene networks and enrichment analyses of *CEL* genes. **(A)** Co-expression genes of *CEL* were analyzed by *Pearson* test based on LinkedOmics database. **(B)** GO and KEGG analysis of *CEL*-related co-expression genes in PDAC. **(C)** The top 50 genes with a positive correlation with *CEL* were visualized in a heatmap. **(D)** The top 50 genes with a negative correlation with *CEL* were visualized in a heatmap. GO, Gene Ontology; KEGG, Kyoto Encyclopedia of Genes and Genomes; PDAC, pancreatic ductal adenocarcinoma.

The above results indicated that *CEL* was significantly associated with CP and PDAC. The interaction between *CEL* gene and its co-expressed genes in pancreatic cancer was then explored. There were 1920 genes (red dots) positively related to *CEL* in PDAC, and 208 genes (green dots) negatively related (false discovery rate (FDR) < 0.01) (Figure 6A; Supplementary Table S2). DAVID was used to analyze GO and KEGG pathways related to these co-expressed genes. The results showed significant enrichment of the biological process (BP) terms “digestion,” “regulation of hormone secretion,” and “regulation of peptide hormone secretion”. The enriched cellular component (CC) terms mainly included “transmembrane transporter complex,” “basolateral plasma membrane,” and “ion channel complex.” The enriched molecular function (MF) terms mainly included “metal ion transmembrane transporter activity,” “monovalent inorganic cation transmembrane transporter activity,” and “sodium ion transmembrane transporter activity.” The KEGG pathway analysis showed that the co-expressed genes were mainly related to “pancreatic secretion,” “protein digestion and absorption,” and “maturity onset diabetes of the young” (Figure 6B). As can be seen, we found that the biological functions of co-expressed genes with *CEL* were basically consistent with the above 85 overlapping DEGs data. Figures 6C, D showed the top 50 positively and the top 50 negatively co-expressed genes associated with *CEL*, respectively.

### Construction of common miRNA gene network

**FIGURE 7:**
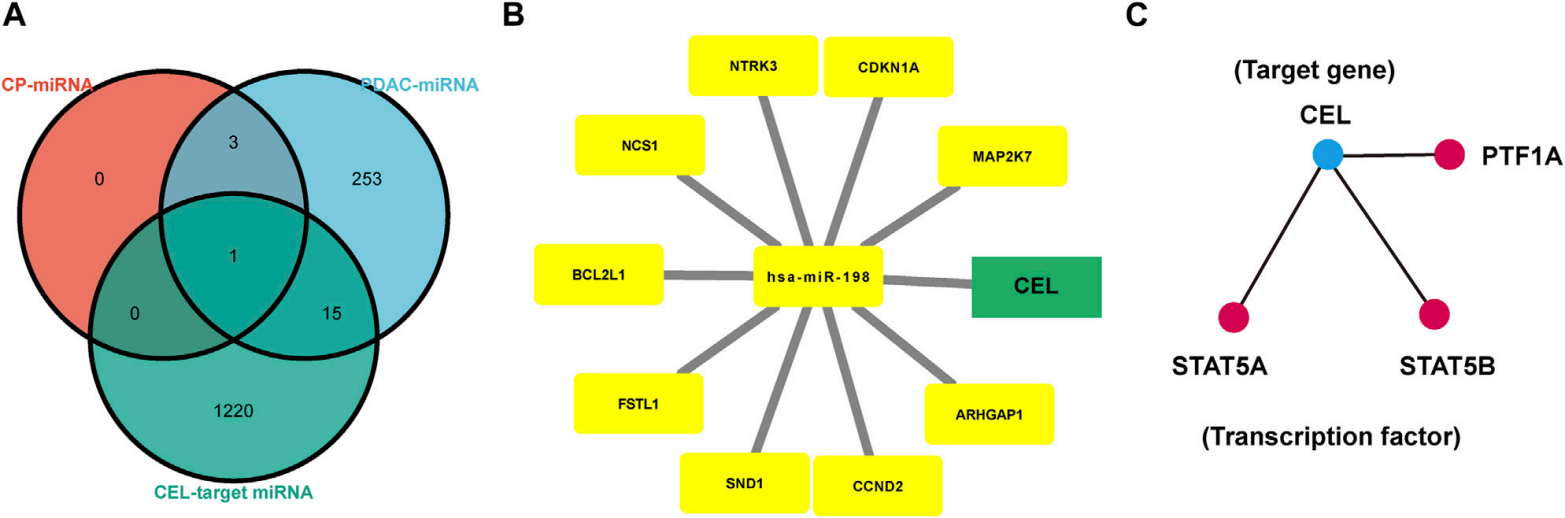
Analysis of TF-miRNA-Hub Gene Network. **(A)** Venn diagrams were recruited to obtain the commoniy predicted miRNAs. **(B)** miRNAs-hub genes regulatory network. Green rectangle represented real hub gene. **(C)** TFs regulatory network. TFs were marked in red, and the hub gene was marked in blue. TF, transcription factors.

The HMDD database was used to identify miRNAs associated with CP and PDAC, respectively. Subsequently, *CEL*-target miRNAs were predicted. The Venn diagram was applied to obtain the miRNA intersection of *CEL*, CP and PDAC (Supplementary Tables S3–S5). The hsa-miR-198 was the intersection of these three miRNA microarray sets (Figure 7A). With the assistance of miRWalk, miRNA-target gene interactions were predicted (Figure 7B). The *ARHGAP1*, *SND1*, *CDKN1A*, *NCS1*, *CCND2*, *NTRK3*, *BCL2L1*, *FSTL1*, *MAP2K7* and *CEL* were potential target genes of hsa-miR-198. The TRRUST database indicated that 3 TFs (STAT5A, STAT5B and PTF1A) may regulate the expression of *CEL* gene (Figure 7C).

### Characterization of the immune cell infiltration landscape

**FIGURE 8:**
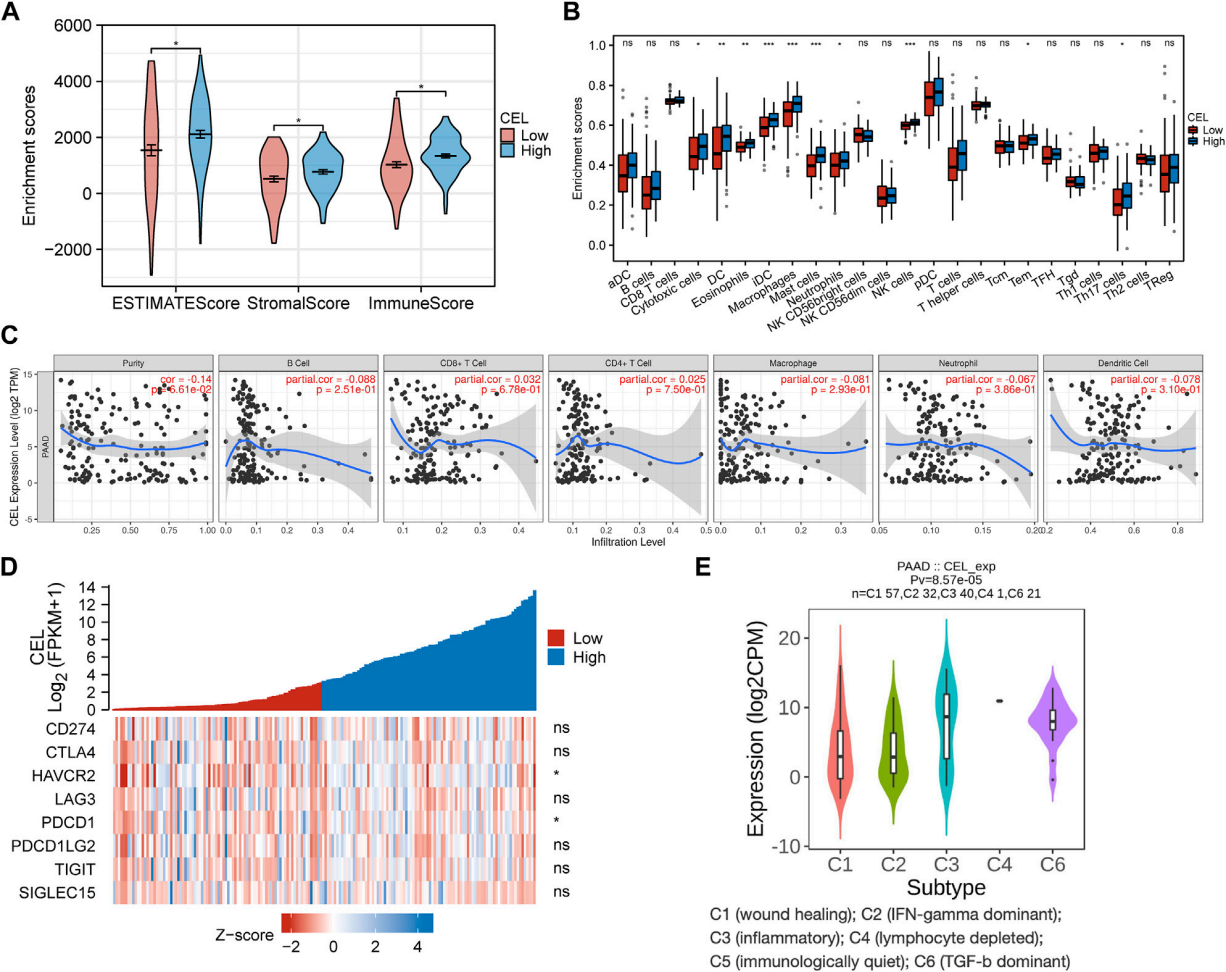
Immune analysis of *CEL* in PDAC. **(A)** Comparing *CEL*-high and *CEL*-low groups based on ESTIMATE scores, stromal scores, and Immune scores. **(B)** Comparison of immune cell subsets between *CEL*-low and *CEL*-high groups. **(C)** *CEL* expression is correlated with immune infiltration in PDAC as analyzed using the TIMER database. **(D)** Heatmap of 8 immune checkpoints between *CEL*-low and *CEL*-high groups. **(E)** *CEL* expression in different immune subtypes of PDAC. C1, wound healing; C2, IFN-gamma dominant; C3, inflammatory; C4, lymphocyte depleted; C5, immunologically quiet; C6, TGF-b dominant. PDAC, pancreatic ductal adenocarcinoma; ns, no significance. ∗*p* < 0.05; ∗∗*p* < 0.01; and ∗∗∗*p* < 0.001.

The EstimateScore, StromalScore, and ImmuneScore were assigned to each PDAC sample according to the estimation algorithm. In the low-*CEL* group, EstimateScore, ImmuneScore, and StromalScore were less than in the high-*CEL* group (*p* < 0.05) (Figure 8A). Moreover, we found that cytotoxic cells, DC cells, eosinophils, iDC, macrophages, mast cells, neutrophils, NK cells, Tem, and Th17 cells were increased in the high-*CEL* group, while the opposite result was found in the low-*CEL* group (Figure 8B). It was examined whether *CEL* influenced immune infiltration in PDAC using the TIMER database. Remarkably, *CEL* expression was positively correlated with the infiltration of CD8^+^ (*p* = 6.78e-01) cells and CD4^+^ (*p* = 7.50e-01) cells. In contrast, *CEL* expression was negatively correlated with the infiltration of B cells (*p* = 2.51e-01), macrophages (*p* = 2.93e-01), neutrophils (*p* = 3.86e-01), and dendritic cells (*p* = 3.10e-01) (Figure 8C). As a result of these studies, *CEL* might play a pivotal role in immune infiltration in PDAC. Furthermore, we constructed a heatmap for known immune checkpoint biomarkers, which indicated differential expression of HAVCR2 and PDCD1 between the two groups. However, other immune checkpoints (CD274, CTLA4, LAG3, PDCD1LG2, TIGIT and SIGLEC15) may not involve in the *CEL*-related immune regulations (Figure 8D). Immune subtypes of solid tumors have been divided into six categories including e C1 (wound healing), C2 (IFN-gamma dominant), C3 (inflammatory), C4 (lymphocyte depleted), C5 (immunologically quiet), and C6 (TGF-b dominant) (Thorsson et al., 2018). With the exception of C5, all other PDAC immune subtypes (C1, C2, C3, C4, and C6) were correlated with *CEL* expression in this study (Figure 8E).

**FIGURE 9:**
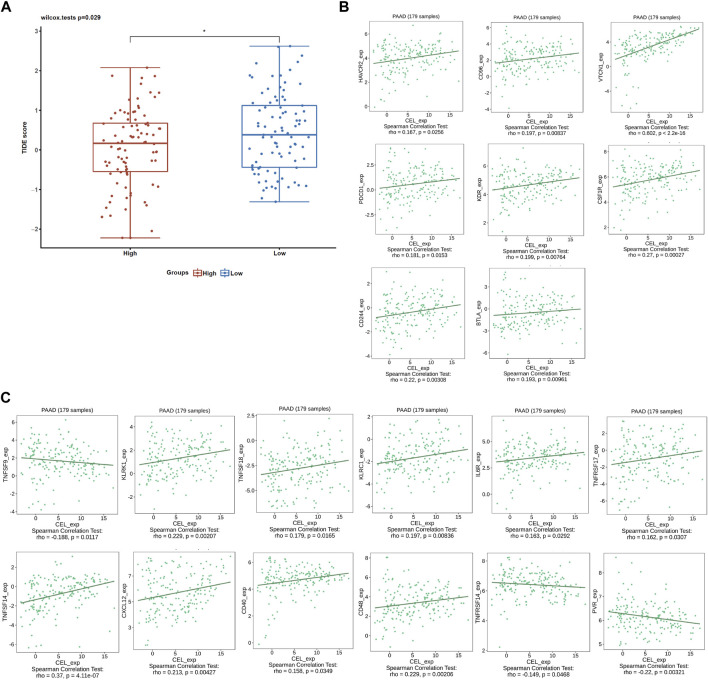
ICB response and immunotherapy. **(A)** Boxplot representation of TIDE scores in the *CEL*-low vs *CEL*-high in TCGA PDAC cohort. **(B)** Correlations between *CEL* expression and immunoinhibitors. **(C)** Correlations between *CEL* expression and immunostimulators.

Given the immune microenvironment is a mediator of ICB responses, we analyzed how that score correlated with the signature of the ICB response. Low expression of *CEL* had lower TIDE scores compared with high expression of *CEL* (Figure 9A). Our results suggested that patients with higher expression of *CEL* were more likely to have greater opportunities of antitumor immune escape. In this study, *Pearson’s* correlation analysis was applied to assess the association of candidate gene expression with immunoinhibitors and immunostimulators. And a total of 8 immunoinhibitors and 12 immunostimulators of *CEL*-expression-related immunomodulators were identified (Figures 9B, C).

**FIGURE 10:**
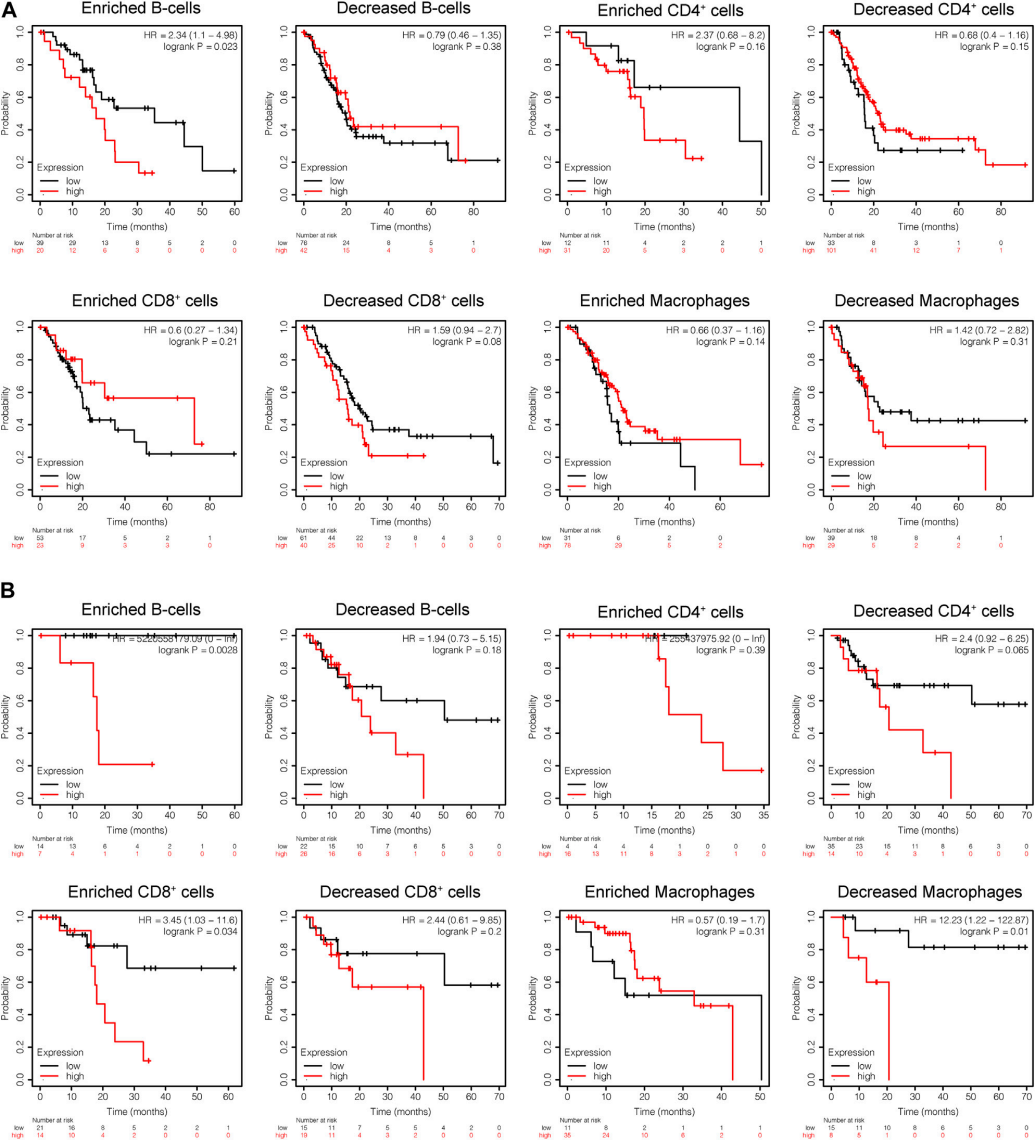
KM analysis of the survival of high and low *CEL* expression groups. **(A)** Relationships between *CEL* of different immune cells subgroup and OS. **(B)** Relationships between *CEL* of different immune cells subgroup and RSF. KM, Kaplan Meier; OS, overall survival; RSF, recurrence free survival.

Subsequently, the prognostic values of *CEL* expression levels for overall survival (OS) and recurrence free survival (RFS) in various immune subgroups of PDAC were further determined with KM analysis. PDAC patients with low *CEL* expression had a favorable OS among enriched B cells, CD4^+^ cells, and decreased CD8^+^ cells (Figure 10A). Low *CEL* expression had a favorable RFS in all immune cell subgroups (Figure 10B). According to the analysis, immune infiltration may influence cancer patient prognoses in part due to low expression of *CEL* in PDAC. However, most of them did not show a statistically significant difference.

## Discussion

PDAC is a highly aggressive lethal malignancy with a poor prognosis due to the lack of early diagnosis and limited response to treatments (Klein, 2021). CP is a progressive inflammatory disease which results in the destruction of the acinar cells and the formation of significant pathologic fibrosis. CP is one of the risk factors for the development of PDAC, however, there is also the possibility that CP may arise from PDAC (Roberts et al., 2016). Since 1993, Lowenfels et al. (Lowenfels AB et al., 1993) reported that patients with CP had a standardized incidence ratio of 26.3 for pancreatic cancer. Based on a pooled analysis within the PanC4 consortium, 6% of PDAC patients reported CP as a complication of their disease (Duell et al., 2012). Through pancreatitis mouse models, some research teams have proven that CP was indispensable for the induction of PDAC (Chen et al., 2017; Takahashi et al., 2021). However, the underlying molecular mechanism of complex interaction between CP and PDAC is still unclear. This research intends to make an initial investigation on the hub gene and signatures of CP and PDAC by comorbidity bioinformatics analysis to improve early detection, treatment, and prevention.

Functional investigations by blasting the genetic data against public databases may reveal the genetic determinants of CP and PDAC. As a result, we are able to get clusters through network analysis and biological processes via GO and KEGG enrichment analysis. Biological processes detoxification of copper ion, cysteine and methionine metabolism, response to hyperoxia, protein digestion and absorption, organ or tissue specific immune response, fat digestion and absorption, and long-chain fatty acid transport were highly enriched in the genes shared by the CP and PDAC groups. According to the PPI analysis, the overlapping DEGs between the two groups were also primarily involved in the transport of nutrients and trace elements such as protein digestion and absorption, detoxification of copper ion, long-chain fatty acid transport, and so on. Considering the results from our study, these pathways could converge into metabolism-related pathways that involve in CP and PDAC. The pancreas is an important retroperitoneal organ, providing both endocrine and exocrine functions. The role of the pancreas for digestion is extremely crucial. CP is characterized by an irreversible damage to both the exocrine (impairment of digestion due to a lack of digestive enzymes produced by the pancreas) and endocrine (mainly owing to the loss of the islets of Langerhans) compartments of the pancreas with progressive inflammation and fibrosis (Singh et al., 2019). An initial event leading to chronic pancreatitis is the premature activation of pancreatic proteases inside the pancreas. And the overactivation of pancreatic proteases may be an important mechanism for triggering and aggravating pancreatic injury. As chronic pancreatitis progresses, the cells that secrete digestive enzymes are gradually destroyed and the anatomy of the pancreatic duct changes (Beyer, et al., 2020). These above-mentioned factors will lead to metabolic dysfunction and dyspepsia in CP patients. PDAC and CP share overlapping clinical symptoms since these two entities have similar histologic features, such as increased intrapancreatic duct pressure, immune cell infiltration, and intense fibrosis. According to these findings, CP and PDAC might be associated with the transport dysfunction of nutrients and trace elements caused by the destruction of acinar cells. These pathological processes were consistent with the GO/KEGG functional enrichment analysis.

Cystic fibrosis transmembrane conductance regulator (CFTR) is the most important molecule for proper pancreatic duct function. In the pancreas, CFTR is an anion channel that secretes bicarbonate to flush digestive enzymes secreted by the acinar cells out of the pancreas. This is a key link in the transportation of nutrients and trace elements. It has been demonstrated that CFTR dysfunction significantly increases the risk of CP (Berke et al., 2022; Phadke and Sellers, 2022). Meanwhile, mutations in CFTR are associated with a modest increase in risk for PDAC (McWilliams et al., 2010; Hennig et al., 2019). As a result, the pathogenesis of CP and PDAC may share common genes and regulators in the dysfunctional transport of nutrients and trace elements.

Through the DEGs analysis of two validated cohorts, 6 shared genes of CP and PDAC were validated to identify the real hub gene. The most exciting finding of this study is the vital role of *CEL* in the progression of CP and PDAC. *CEL* expression was predominantly detected in pancreatic acinar cells and lactating mammary glands in humans (Dalva et al., 2017). As a digestive enzyme, *CEL* is naturally produced and secreted in the acinar cells as a component of pancreatic juice (Lombardo et al., 2017). Once activated by bile salts, this digestive enzyme plays a role in the hydrolysis and absorption of cholesterol and lipid-soluble vitamins. In addition to being present in the intestinal lumen, *CEL* also participates in the metabolism of lipoproteins and atherosclerosis by acting in the circulation (Loli et al., 2015). There are 11 exons in the human *CEL* gene, which spans approximately 10 kb on chromosome 9q34.3 (Johansson et al., 2018). The *CEL* variants are best known for causing maturity-onset diabetes in the young (MODY8). One of the characteristics of MODY8 is characterized by reduced secretion of the protein product (Kahraman et al., 2022). Miyasaka et al. (Miyasaka et al., 2005) found that the increase of the length of variable number of tandem repeats (VNTR) in *CEL* is a risk factor for alcohol induced CP. However, due to ethnic differences or methodological problems, an opposite result was obtained in some cohorts of European descent (Ragvin et al., 2013; FjeldJ et al., 2016). There is a common single uucleotide polymorphisms (rs488087) present in the second repeat of the *CEL* VNTR that is associated with an increased risk of PDAC. Studies have shown that *CEL* variants could be identified using specific antibodies (Johansson et al., 2018). These studies indicate that *CEL* has could be a novel diagnostic marker in CP and PDAC patients. Consistent with the previous study, our study demonstrated that *CEL* expression was significantly decreased when pancreatic neoplastic cells acquired a ductal phenotype (El Jellas et al., 2018).

To understand the relevance of *CEL* expression in PDAC, a further analysis was conducted to investigate whether the *CEL* expression is correlated with the TCGA clinical-pathological parameters. From the TCGA database, PDAC patients were categorized into *CEL* low- and high-expression groups. We found *CEL* level was associated with N stage, pathologic grade and anatomic neoplasm subdivision in the PDAC patients. These results indicated that *CEL* promoted pancreatic cancer cell metastasis. Cancers frequently display genic variations and may contribute to tumor progression. It was proposed that several structural variants of the *CEL* locus, such as protein misfolding, had some effect on pancreatic malignant tumorigenesis (El Jellas et al., 2018; Dalva et al., 2020). Among them, *CEL-HYB* and *CEL-MODY* in the structural variant type play leading roles in pancreatic disease development. Our study also demonstrated the diagnostic value of *CEL* in PDAC patients with a worse prognosis. Further the LinkedOmics database study showed that *CEL* promoted digestion, transmembrane transporter complex, metal ion transmembrane transporter activity, pancreatic secretion, and so on. Based on these results, *CEL* is the crossroads of the clinical and pathological signaling pathways for both CP and PDAC.

A miRNA is a small non-coding RNA molecule (21–25 nucleotides long) that induces the degradation of a target gene mRNA by binding to the 3′URT of the target gene mRNA (Sharma et al., 2019). Post-transcriptional regulation of miRNA has been extensively demonstrated to be involved in the development of various diseases (Correia de Sousa et al., 2019). Consequently, we constructed a relational network between miRNAs and candidate target genes based on the HMDD database and miRWalk. As a result of the intersection of three miRNA microarray sets, hsa-miR-198 was identified. Tumor biomarkers based on miRNA expression profiles can be detected at low levels. It has been shown that hsa-miR-198 is able to differentiate CP from PDAC (Vychytilova-Faltejskova et al., 2015). In addition, the hsa-miR-198 can act as a tumor suppressor depending on the type of cancer (Kaushik and KumakR, 2022). As a result, we speculate that hsa-miR-198 may contribute to PDAC pathophysiological development through inflammatory processes. The transcription factor (TF) binds to cis-regulatory elements in DNA and activates RNA polymerase to regulate gene transcription. Therefore, identifying the interaction pattern between TFs and their target genes is vital for biological and medical research. In our study, STAT5A, STAT5B and PTF1A were predicted to function as a regulatory TF upstream of *CEL* based on regulatory associations revealed in the TRRUST database. Among them, STAT5A and STAT5B, were encoded by separate genes and induced by Janus-activated kinases (JAK) in the mitochondria (Buchert et al., 2016). STAT5 family protein strictly regulates cell proliferation and differentiation and plays a pivotal role in maintaining normal immune function and body balance (Rani and Murphy, 2016). A previous research reported an abnormal phosphorylation of STAT5 occurred during the transformation from pancreatic inflammation to PDAC (Juusola et al., 2021). The transcription factor PTF1A is a crucial helix-loop-helix (bHLH) protein that controls the expansion of pluripotent progenitor cells and the development and maintenance of acinar cells (Jin and Xiang, 2019; Duque et al., 2022). Isolated pancreatic aplasia can be caused by some hypomorphic PTF1A mutations (Houghton et al., 2016). Moreover, the downregulation of PTF1A has also been associated with pancreatic intraepithelial neoplasia (Krah et al., 2015). It may be possible to treat PDAC with small molecule drugs that promote PTF1A upregulation. In this study, we provided potential directions for clarifying the molecular mechanism of PDAC progressed by CP.

TIME has been shown to promote tumor development, progression, and immunoevasion (Barker et al., 2015; Itoh et al., 2018; Piao et al., 2018). We revealed a negative correlation of the expression of *CEL* with the infiltration of immune cells (B cells, macrophages, neutrophils, and dendritic cells) in PDAC. An important function of B cells in adaptive immunity is to produce antibodies. B cells activated in tumors can further activate T lymphocytes, potentiating antitumor immunity (Germain et al., 2015). However, the roles of B lymphocytes in tumors were complex, and sometimes B cells can suppress antitumor immune responses (Yuen et al., 2016). There were several studies confirming that the depletion of B cells suppressed pancreatic tumorigenesis (Lee et al., 2016; Tspu et al., 2016). In our study, the low expression of *CEL* was associated with better prognosis in the PDAC patients with B-cell enrichment. Macrophages play a pivotal immune role in inflammatory and malignant diseases (Chupradit et al., 2022; Pittet et al., 2022). In the PDAC stroma, there is a macrophage type called tumor-associated macrophage (TAM) that is more inclined to polarize in the M2 cell type (Pratt et al., 2021). In pancreatic intraepithelial tumors, TAMs are one of the first infiltrating cells, and their numbers increase persistently during cancer progression (Beatty GL et al., 2017; Bear et al., 2020). A previous study has confirmed that macrophage density was an independent prognostic factor of PDAC, which was associated with the risk of disease progression, recurrence, metastasis, and shorter overall survival (Yu et al., 2019). In the present study, the decreased macrophages have been linked to improved survival for PDAC patients with low *CEL* expression. In addition, higher CD4^+^ helper T-cell densities were associated with improved survival among PDAC patients, but not with higher CD8^+^ helper T-cell densities (Dias Coasta et al., 2022). As our results indicate, CD4^+^ T-cell expression increases with decreased *CEL* expression. PDAC patients with low *CEL* expression have been shown to benefit from the desert zone of CD4^+^ T-cell. Dendritic cells (DCs) are key mediators of adaptive immunity, playing a central role in initiating antigen-specific immunity (Kvedaraite and Ginhoux, 2022). In spite of the fact that DCs are essential for immune activation and effector cell recruitment, it has been reported that tumor cells secrete the immunosuppressant cytokine IL-10, which inhibits DC maturation or converts DCs into macrophage-like cells (Gajiwala et al., 2018; Pu et al., 2018). In addition, a positive correlation was found between *CEL* expression and corresponding immune cell markers and immune checkpoints. Meanwhile, we also identified immunoinhibitors and immunostimulators with therapeutic effects on *CEL* expression for PDAC patients. Ultimately, these results confirmed that *CEL*, a real hub gene for both CP and PDAC, played a significant role in TIME.

There are several limitations to this study, such as the insufficient number of databases included. In addition, this study is only a bioinformatics analysis of *CEL* function in PDAC. The role of *CEL* in transforming chronic inflammation into PDAC may be confirmed in future basic research.

## Conclusion

**FIGURE 11:**
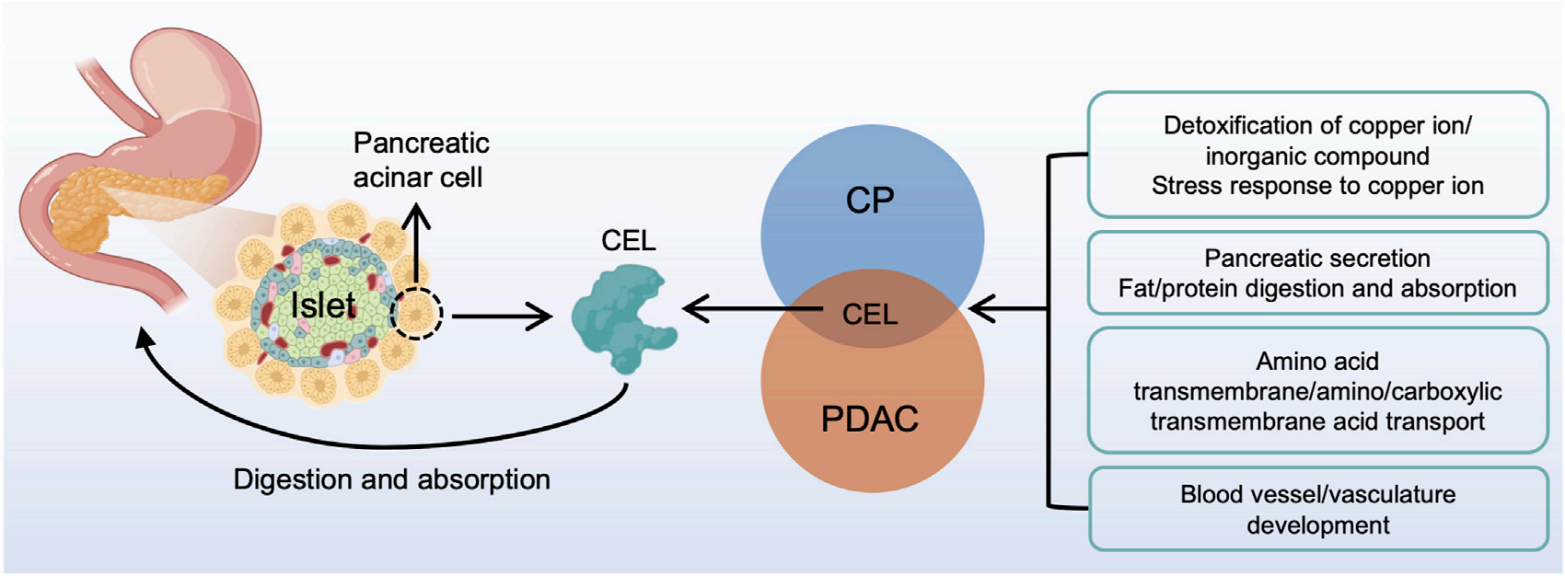
A brief overview of CP and PDAC interactions. *CEL* played a central role in the interactive development of CP and PDAC. The common characteristic of CP and PDAC patients were protein digestion and absorption, detoxification of copper ion, long-chain fatty acid transport and vasculature development. CP, chronic pancreatitis; PDAC, pancreatic ductal adenocarcinoma.

In summary, our study identified the real hub gene and its signatures potentially related to CP and PDAC (Figure 11). The common characteristics of CP and PDAC patients were mainly protein digestion and absorption, detoxification of copper ion, long-chain fatty acid transport and vasculature development. There was some evidence that the metabolism-related pathways in CP might be an essential factor for the development of PDAC. Examination on these common pathways and real hub genes may shed light on the molecular mechanism underlying CP and PDAC development.

## Data Availability

The datasets presented in this study can be found in online repositories. The names of the repository/repositories and accession number(s) can be found in the article/Supplementary Material.
