# Supplementary material for: Exploring the key genetic association between chronic pancreatitis and pancreatic ductal adenocarcinoma through integrated bioinformatics
Source: Front Genet. 2023 Jul 12;14:1115660. doi: 10.3389/fgene.2023.1115660 (PMC10369079; doi:10.3389/fgene.2023.1115660)
Supplement: Supplementary file 3 [file Table1.docx]

| Characteristic | Low expression of *CEL* | High expression of *CEL* | *p* |
| --- | --- | --- | --- |
| N | 89 | 89 |  |
| Age, mean ± SD | 64.6 ± 10.61 | 64.9 ± 11.04 | 0.852 |
| Gender, n (%) |  |  | 1.000 |
| Female | 40 (22.5%) | 40 (22.5%) |  |
| Male | 49 (27.5%) | 49 (27.5%) |  |
| T stage, n (%) |  |  | 0.277 |
| T1 | 6 (3.4%) | 1 (0.6%) |  |
| T2 | 12 (6.8%) | 12 (6.8%) |  |
| T3 | 69 (39.2%) | 73 (41.5%) |  |
| T4 | 1 (0.6%) | 2 (1.1%) |  |
| N stage, n (%) |  |  | 0.010 |
| N0 | 33 (19.1%) | 17 (9.8%) |  |
| N1 | 53 (30.6%) | 70 (40.5%) |  |
| M stage, n (%) |  |  | 0.650 |
| M0 | 34 (40.5%) | 45 (53.6%) |  |
| M1 | 3 (3.6%) | 2 (2.4%) |  |
| Pathologic stage, n (%) |  |  | 0.007 |
| Stage I | 17 (9.7%) | 4 (2.3%) |  |
| Stage II | 66 (37.7%) | 80 (45.7%) |  |
| Stage III | 1 (0.6%) | 2 (1.1%) |  |
| Stage IV | 3 (1.7%) | 2 (1.1%) |  |
| Anatomic neoplasm subdivision, n (%) |  |  | 0.020 |
| Head of Pancreas | 62 (34.8%) | 76 (42.7%) |  |
| Other | 27 (15.2%) | 13 (7.3%) |  |

**Table S1.** Clinical pathological parameters of the PC patients with tumors expressing high or low *CEL* in TCGA database.
