# Supplementary material for: Exploring the key genetic association between chronic pancreatitis and pancreatic ductal adenocarcinoma through integrated bioinformatics
Source: Front Genet. 2023 Jul 12;14:1115660. doi: 10.3389/fgene.2023.1115660 (PMC10369079; doi:10.3389/fgene.2023.1115660)
Supplement: Supplementary file 4 [file Table4.DOCX]

**Supplementary Information**


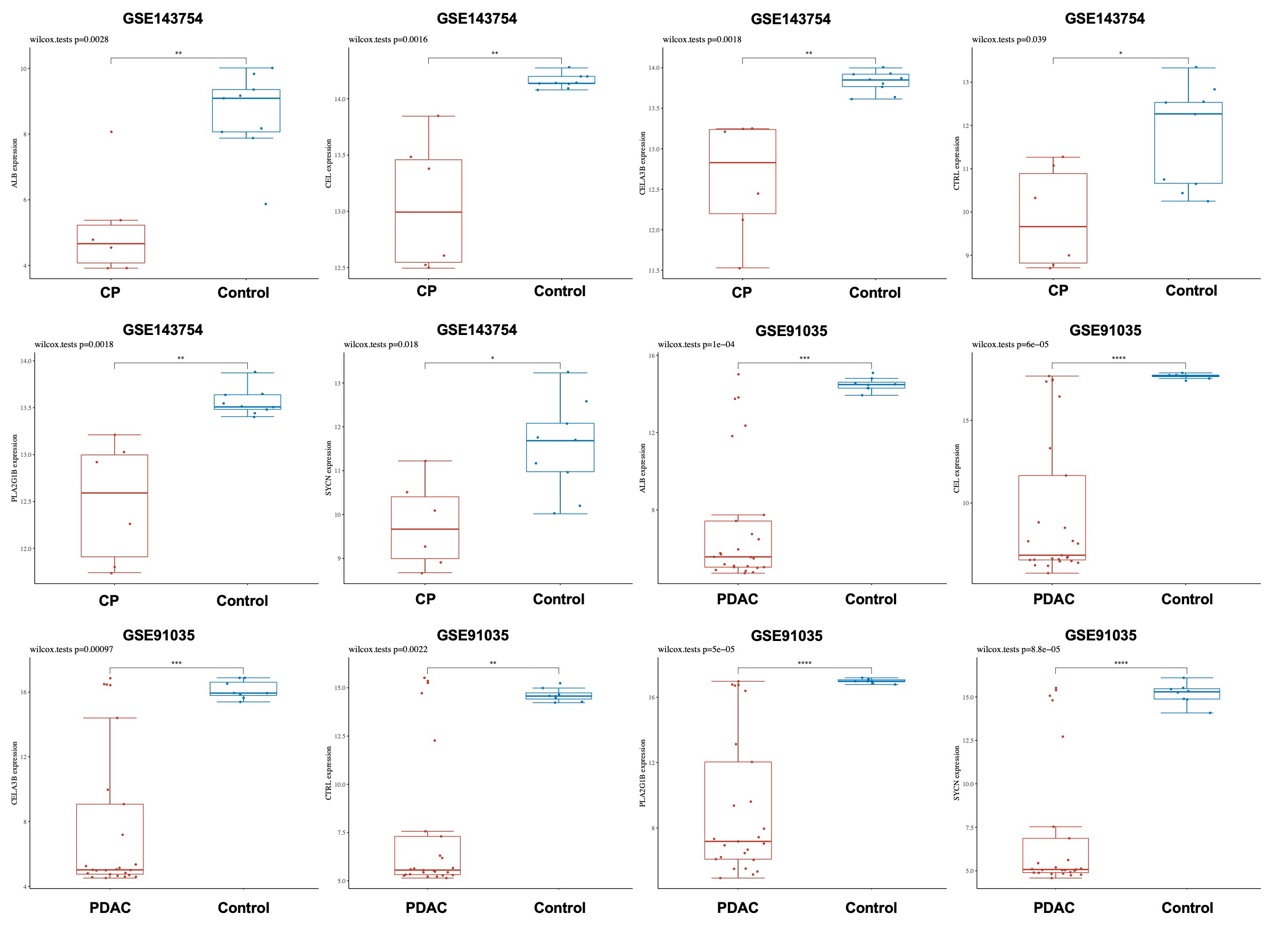


**Figure S1:** Boxplots of the expression of real hub genes in discovery sets.
